# Facile and inexpensive fabrication of zinc oxide based bio-surfaces for C-reactive protein detection

**DOI:** 10.1038/s41598-018-30793-z

**Published:** 2018-08-23

**Authors:** Lu Cao, Janice Kiely, Martina Piano, Richard Luxton

**Affiliations:** Institution of Bio-sensing Technology, University of the West of England, Frenchay Campus, Bristol, BS16 1QY UK

## Abstract

The paper reports a biosensor formed from antibody coated ZnO nano-crystals which has been prepared using a rapid and inexpensive fabrication method which utilises colloidal dispersion enhanced using sonication. This technique was used to prepare highly ordered and uniform nano-crystalline sensor surfaces on polyethylene terephthalate (PET) using 0.5%, 1% and 5% concentrations of zinc oxide nano-crystal suspensions. Impedance spectroscopy was employed to interrogate the sensor surfaces and confirmed high reproducibility of the fabrication process. Changes in impedance values, at a frequency of 138 Hz, were used to establish dose dependent responses for C-reactive protein (CRP) antigen. A limit of detection of less than 1 ng/ml was demonstrated for nano-surfaces fabricated from concentrations of 1% ZnO.

## Introduction

Nanomaterials have been intensively studied and provide an excellent platform for the development of high performance biosensors, due to their unique physio-chemical properties. For example, due to the large surface area of nanomaterials, large numbers of capture molecules, such as enzymes^1–3^, antibodies^4^, and DNA^5^, can be immobilised, enhancing sensitivity. Zinc oxide (ZnO) nanoparticles are one of the most important nanomaterials in this context, due to their high electron mobility, good chemical stability, low toxicity and biological compatibility^6^. Furthermore, ZnO has a high isoelectric point (pI) of approximately 9.5, which makes it suitable for absorption of relatively negatively charged proteins, e.g. enzymes and antibodies with lower pI’s, primarily driven by electrostatic interaction. The majority of reported ZnO-based biosensors are designed for the detection of various molecule analytes by electron transfer, such as glucose^3,7,8^, cholesterol^9,10^, cortisol^11^, cardiac troponin (cTnT)^12^ and phenol^13^. It has been used for detection of pentachlorophenol (PCP) via measuring electrochemiluminescence (ECL) intensity at electrode surface caused by electrochemical high-energy electron transfer reaction^14^. ZnO also has been used to fabricate electrochemical impedance sepectroscopy (EIS) based sensors for detection of glucose^8^. Furthermore, EIS has also been shown to indicate the effect of different cTnT concentrations on the charge perturbations at the electrode surfaces^12^.

From a structural perspective, zinc oxide crystallizes in two main forms, hexagonal wurtzite and cubic zinc blende, the wurtzite structure is most stable at ambient conditions and thus most common. ZnO can produce a piezoelectric effect once it is strained due to the non-centrosymmetric crystal structure^15^. The piezoelectric constant is strongly sensitive to both temperature and stress^16^. Because of the role of native point defects and impurities, innately ZnO has an n-type conductivity^17^. A number of methods have been used to synthesize ZnO films and other types of nanostructures for biosensors, such as chemical baths^18^, hydrothermal methods^19^, chemical vapour deposition(CVD)^20^, Pulsed Laser Deposition(PLD)^11^, sputtering^21^, electro-spinning^2^ and sweeping-printing^22^. However, the preparation of these films and structures, such as nanowires^18^, nanorods^4,9,21^, nanofibres^2^ and nanotubes^1,23^, with desirable electrical/chemical properties remains a technological challenge. One example of the complexity of achieving high quality, reproducible structures is described by Sanguino^24^. They used hydrothermal process to gain a high density of ZnO structures deposited on Au microelectrodes, however, the lack of deposition time caused the uneven coverage of the electrode^24^.

Compared with other methods, the technique of colloidal dispersion of ZnO nanoparticles shows both disadvantages and advantages. The disadvantage of ZnO nano-crystals is that the crystals easily aggregate. During the process of growth, Park *et al*.^25^ found that several ZnO crystallites of about 5–10 nm agglomerated and formed a horizontal hexagonal platelet, which was a different output compared with other published results. Through a comparison of different concentrations (1%, 3%, 5% and 7%) of ZnO dispersions either in water or methanol to make the surface of ZnO-polyester composite textile materials, Rimbu *et al*.^26^ demonstrated that the larger concentration leads to agglomerations and a diminished coating quality. In order to mitigate the problem of aggregation, the process of sonication can be used. For example, in order to fabricate an ultrasensitive biosensor for DNA detection, Liu *et al*.^5^ employed sonication to achieve water-soluble ZnO/Au nanocomposites. The advantage of the colloidal dispersion techniques is that ZnO nanocrystals of defined shape and size can be purchased ensuring that well characterised films can be created via a simple fabrication process.

In this study, we present preliminary results of a new biosensor that utilises a colloidal dispersion technique, incorporating sonication, to create a ZnO nanocrystal surface. The advantage of this technique is that biosensor surfaces, with controlled compositions and nanostructures, could be created at low cost with standard laboratory equipment; the technique also being suitable for large scale synthesis. Impedance spectroscopy was used to interrogate the biosensor surface to create a compact biosensor. In order to prove the functionality of the ZnO nano-crystal biosensor, C-reactive protein (CRP) was selected as the model antigen. CRP has been shown as a biomarker of various health outcomes, such as cardiovascular disease^27^, obesity^28^, diabetes^29^, cerebrovascular disease^30^, chronic kidney disease^31^, cancer^32^.

## Results and Discussion

### Morphological Study

**Figure 1:**
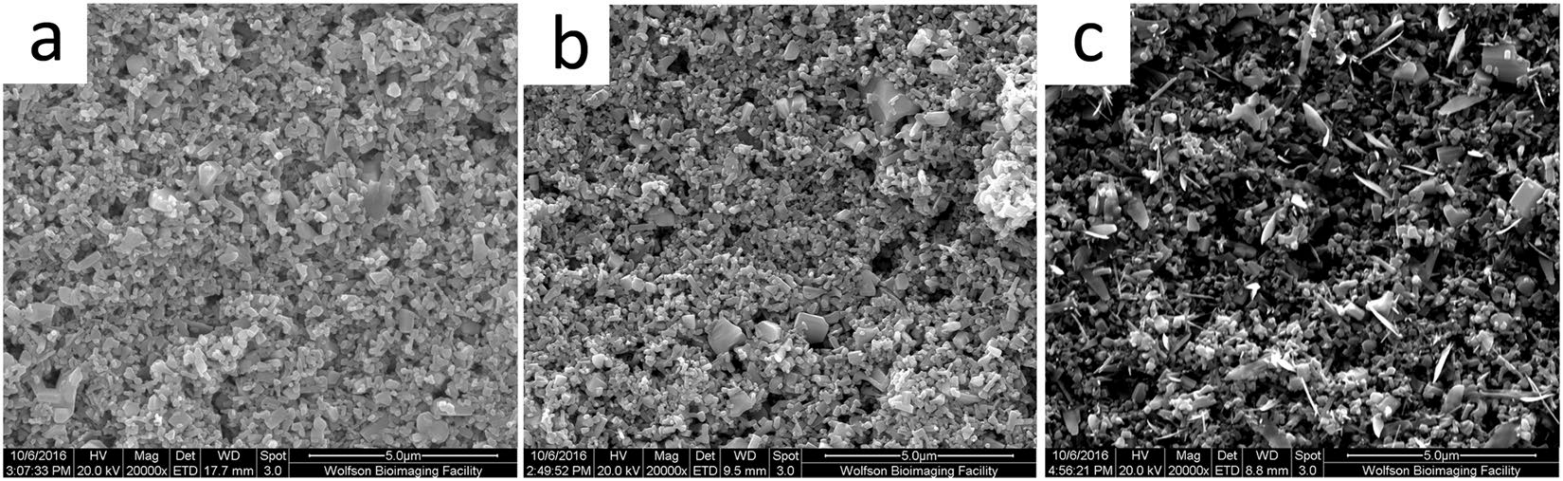
SEM images for ZnO nano-surfaces on PET via suspensions at different amounts of ZnO nano-crystals: (**a**) 5% ZnO; (**b**) 1% ZnO; (**c**) 0.5% ZnO.

From the SEM images, Fig. 1(a), the 5% ZnO suspension shows a surface with a more complete covering of ZnO compared with Fig. 1(b), the 1% ZnO suspension. This observation was as expected owing to the larger mass of ZnO applied. Figure 1(c) shows that the surface of 0.5% ZnO nano-crystals on PET is significantly different from the 1% and 5%, with abnormal sharp, platelet-like shapes. We postulate that the relative absorption of ultrasound energy is greater by the crystals when there is a lower mass of ZnO material (0.5% ZnO), resulting in the fracture of the ZnO crystals forming the observed sharp platelets. This suggests that the size of the composites observed could be due to the sonication-induced aggregation^5^. In other words, the lower concentrations of ZnO suspension are subject to larger sheer forces which may fracture the existing nanocrystal structures and subsequent agglomerations result in the formation of the other ZnO nano-structures observed.

**Figure 2:**
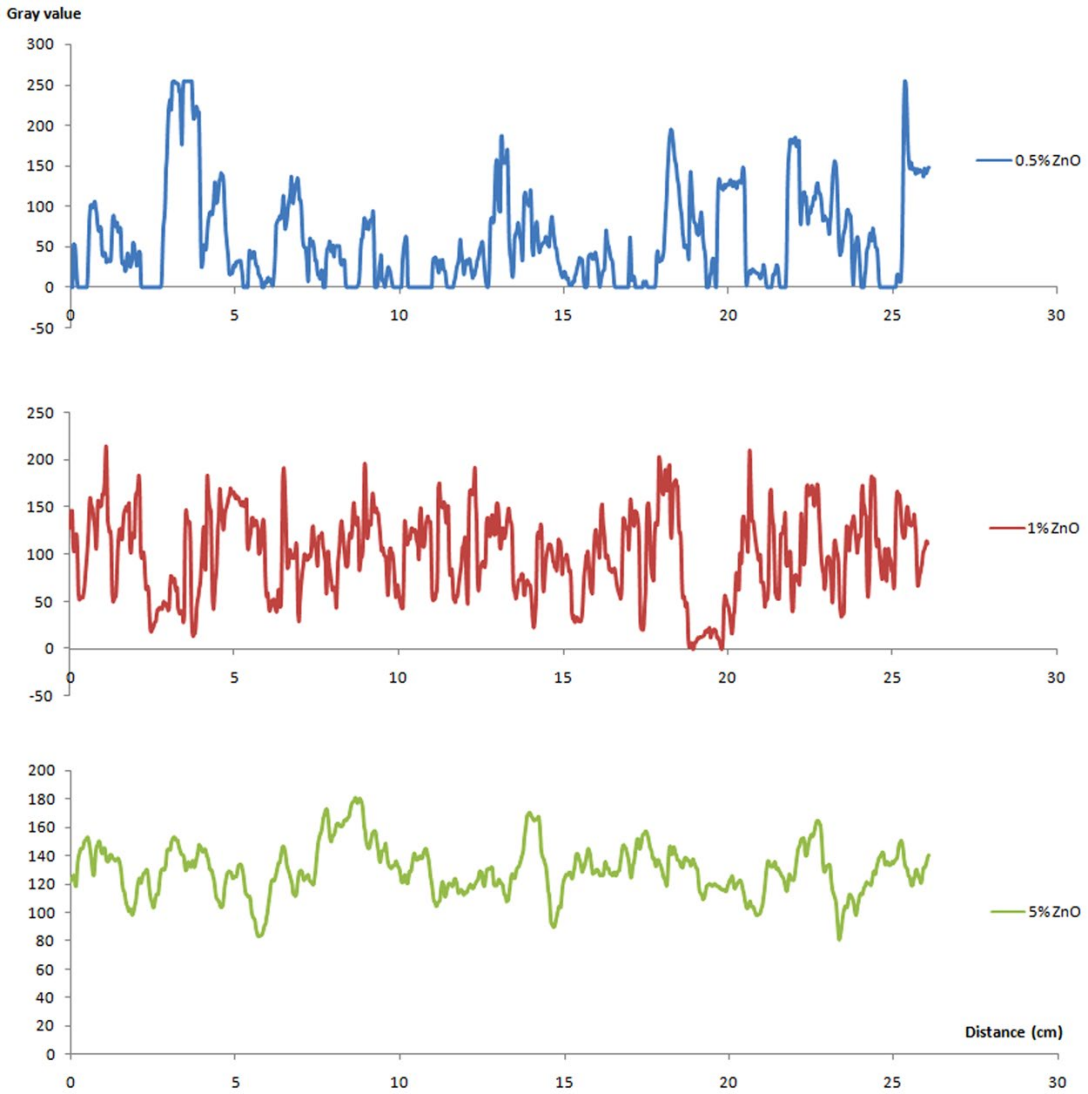
Plot profiles along a horizontal line within images (mag × 2000) for nano-surfaces made by three different concentrations of ZnO suspensions: 0.5%, 1% and 5%.

From Fig. 2 it is clear that even though the surface is covered with dense nanoparticles, there are still many voids resulting in a porous surface. The lower values of grayscale represent darker areas on the image where the substrate is revealed and the higher grayscale values represent the lighter areas with a maximum grayscale value of 255. The profile plot of 5% ZnO shows the narrowest range of grayscale values compared with 1% and 0.5%, indicating that the surface of 5% is smoother than the others, with less deep holes and peaks across the nano-surface, reflecting the high density of ZnO nanocrystals present. This demonstrates that the colloidal dispersion technique and drop method provides uniformed ZnO films with 5% ZnO. In comparison, the 1% ZnO nano-surface gave full coverage with ZnO but with a rougher surface, whereas the 0.5% nano-surface showed many areas where the grayscale value was zero, indicating deep pits, revealing the underlying PET. The roughness index of the 0.5% ZnO nano-surface was calculated to be 8097, the 1% ZnO nano-surface was 10718 and the 5% ZnO nano-surface was 2310. These results indicate that 1% ZnO nano-surface has the largest surface area; the 0.5% ZnO nano-surface had a relatively large surface area but there was incomplete coverage of the PET substrate, whereas the 5% nano-surface is the least rough, with the smallest surface area.

### Raman Spectroscopy analysis of the surfaces

**Figure 3:**
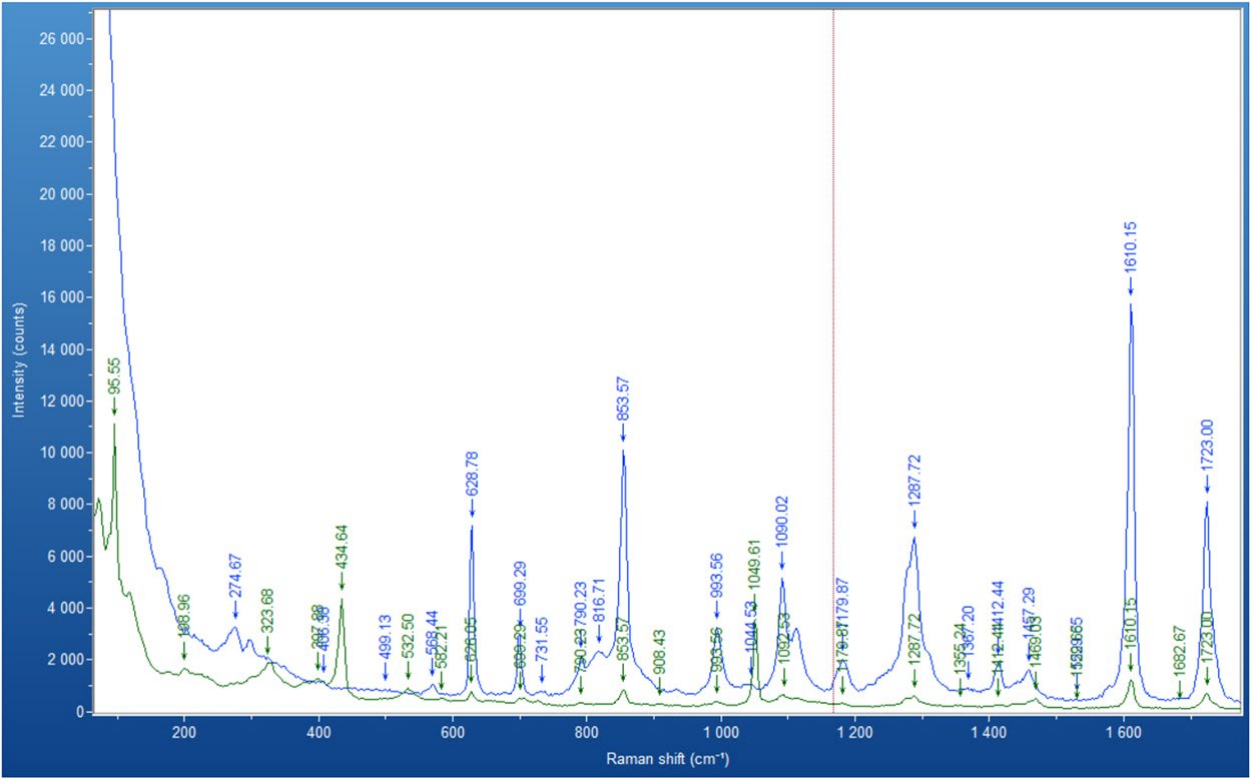
Raman spectra of bare PET (blue line) and PET covered by 1% ZnO nanocrystals (green line) at room temperature. Conditions of recording Raman Spectrum of bare PET: time acquisition 30 s, wavelength 785 nm. Conditions of recording Raman Spectrum of nano-ZnO on PET: time acquisition 50 s, wavelength 785 nm.

Figure 3 shows the Raman spectra of bare PET (blue line) and PET covered with 1% ZnO nanocrystals (green line). There was a strong correlation with a Raman spectrum of pure PET^33^, two strong, characteristic bands were observed at 1723 cm^−1^ and 1610 cm^−1^ which corresponded to C=O stretching and benzene ring structures. The band at 854 cm^−1^ corresponded to the ester C(O)O bending mode^33^. Published Raman spectra of ZnO nanorods^34,35^, indicate significant bands at 438 cm^−1^ and 1050 cm^−1^ and a few weak bands at 330, 379, 535 and 585 cm^−1^. The Raman spectrum of the ZnO surface shown in Fig. 3, shows that the bands associated with the PET are attenuated due to being covered by ZnO, additional bands are seen at 324, 435, 532, 582 and 1050 with the significant bands at 324 cm^−1^ and 1050 cm^−1^. These results strongly indicate the ZnO nano crystals cover the surface. A Raman peak at 324 cm^−1^ represents a highly crystalline structure in ZnO nanomaterials, while peaks at 584 and 673 cm^−1^ represent disordered material and impurities^36^. Comparing the intensity of two peaks at 324 and 582 cm^−1^, the peak at 324 cm^−1^ was far greater, indicating that the nano-surface with ZnO crystals were highly ordered and uniform.

**Figure 4:**
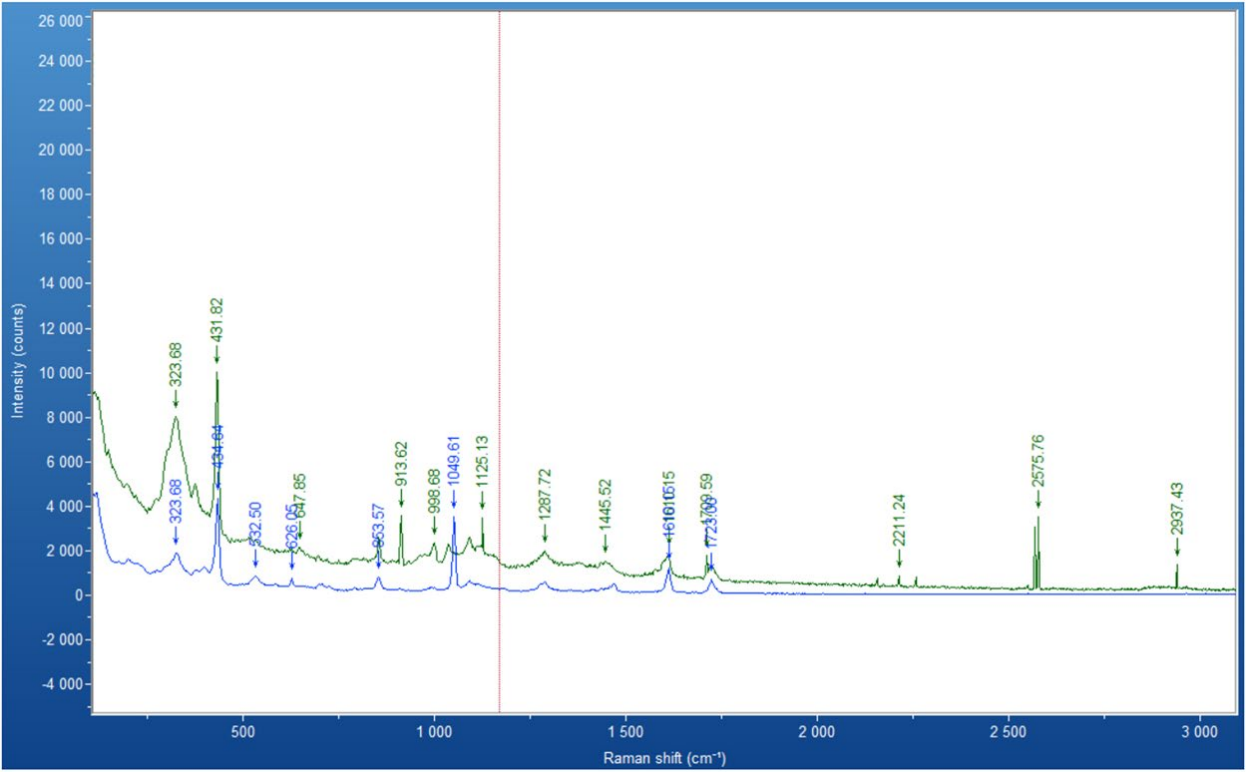
Raman spectra of ZnO nano-surface on PET (blue line) and ZnO with capture antibody on PET (green line). Conditions of recording ZnO with capture antibody on PET: time acquisition 150 s, laser wavelength 785 nm.

Figure 4 shows the Raman spectra for 1% ZnO with capture antibody (green line) and for ZnO without the capture antibody molecule (blue line). The main goal is to identify antibody on the surface of the ZnO through the spectra derived. The anti-Human C-reactive protein, used in this study is an IgG2a isotype. Bands associated with antibody were observed at 2937, 2576, 2211, 1125, 999 and 914 cm^−1^, providing strong evidence that antibody is on the surface of the ZnO nanocrystals. Kengne-Momo^37^ reports that IgG will give bands at 914 cm^−1^ (CH_2_ deformation (*ρ*CH_2_); 2937 cm^−1^ (C-H stretching (*ν*C-H) of aliphatic chains). Both of these bands are evident in Fig. 4. In addition, the backbone skeletal *ν*C-C vibration bands were observed in the region of 999 to 1125 cm^−1^.

### Impedance spectroscopy of the surface of the PET with different concentrations of ZnO

**Figure 5:**
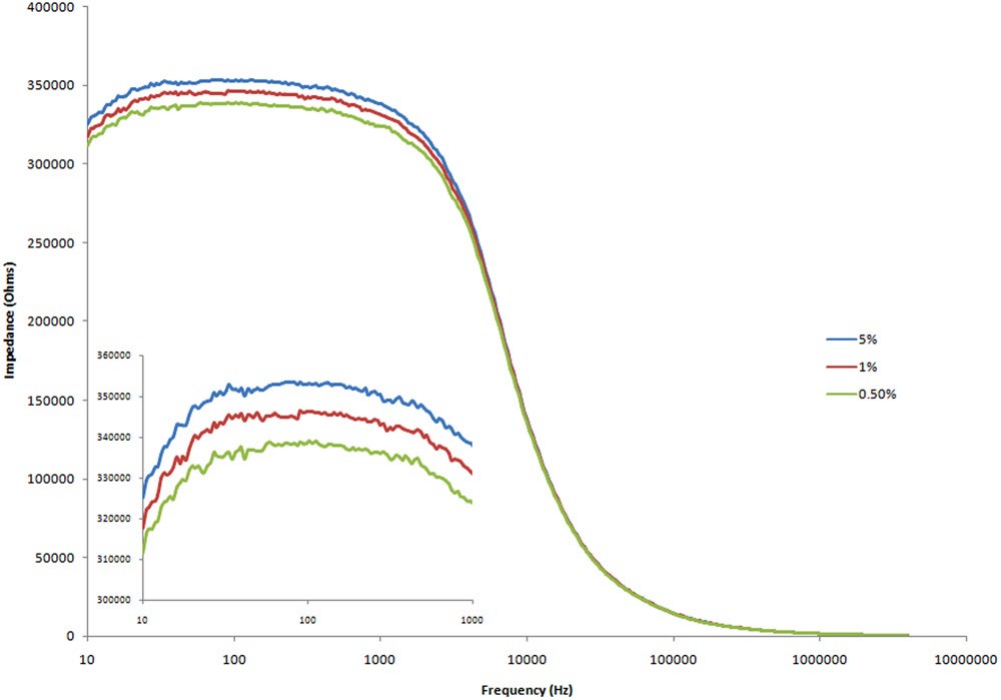
Impedance spectra of three different concentrations of ZnO nano-powders (blue: 5%, red: 1%, lime green: 0.5%) on PET substrates.

Impedance spectroscopy of the three ZnO nano-surfaces (from concentrations of ZnO nano-crystals suspensions of 5%, 1%, 0.5%) are shown in Fig. 5. The results were plotted based on average impedance value of 24 samples for each concentration. These measurements were made on different sensors over a series of days. Fig. 5 shows that the average impedance values decreases as the concentrations of ZnO suspension decreases. This is due to the high loading of zinc oxide in the 5% sample causing the maximum perturbation of the electromagnetic field of the impedance sensor, due to the increased positive charge associated with the ZnO crystals.

The greatest difference between the impedance values of the nano-surfaces at different concentration of ZnO occurred at a frequency of 138 Hz. This frequency was similar to frequencies used in the literature. For example, Jacobs *et al*.^38^ found that the most significant changes occurred around 100 Hz, when adding various concentrations of the protein troponin-T on to a ZnO surface. Jacobs reported that this was due to the fact that the electrical double layer is greater at frequencies below 1000 Hz^38^. A statistical analysis of the impedance values taken at a frequency of 138 Hz is shown in Table 1. The %CVs demonstrate that the fabrication process is reliable as the 24 measurements were performed on individual sensor surfaces. Although all surfaces were highly reproducible, the highest concentration of ZnO nano-crystals resulted in the highest reproducibility. At all concentrations, there was a highly significant difference between the impedance of bare PET material and the impedance of the PET with ZnO (p < 0.001).

**Table 1:** Data analysis on impedance values on each set of ZnO nano-surfaces at fixed frequency.

Different concentrations of ZnO suspensions dropped on PET	N	Mean	StDev	CV(%)
5%	24	352870	2453	0.70
1%	24	345567	4117	1.19
0.5%	24	338387	5633	1.66

### Calibration curve comparison of the three ZnO-PET surfaces with CRP immobilised

**Figure 6:**
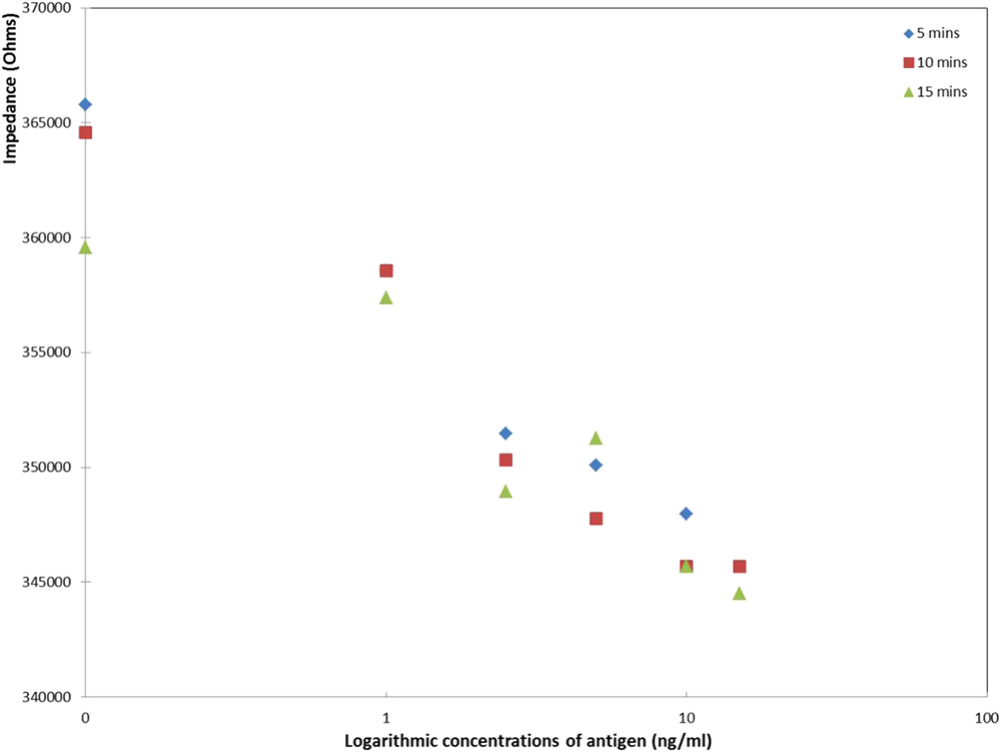
Impedance values from 1% ZnO nano-sensors, with 100 ng capture antibody with increasing concentrations of antigen: 0–15 ng/ml, for 5, 10 and 15 minutes incubation time.

To examine the effect of varying the incubation period, measurements were performed on 1% ZnO nano-surfaces prepared with antibody and incubated for 5, 10, 15 minutes. Figure 6 shows the absolute value of the impedance as a logarithmic function of CRP concentration for each of the 3 nano-surfaces. The plots were similar indicating that beyond 5 minutes the incubation time does not greatly influence the result, which suggests that binding of CRP to the antibody occurs rapidly. The dose dependent response of absolute impedance for each incubation time shows decreasing impedance with increasing CRP loading. This can be explained by the fact that in this experimental set-up the ZnO is positively charge, whereas the CRP is dominated by negative charge (pI of CRP is ~5.45). Electrochemical impedance spectroscopy (EIS) can be used to measured an electrical double layer (EDL) formed when a semiconducting material interacts with liquid electrolytes^11^. Consequently, when binding to the ZnO occurs, through capture by the antibody on the surface, there is a decrease in the overall positive charge with increasing CRP loading and a decrease in the absolute impedance value. This observation concurs with the literature where it is reported that, for non-faradaic biosensors, changes in the surface dielectric and charge distribution are induced when a protein target binds to the receptor, previously attached in the electrode, displacing water and ions from the surface^39^.

**Figure 7:**
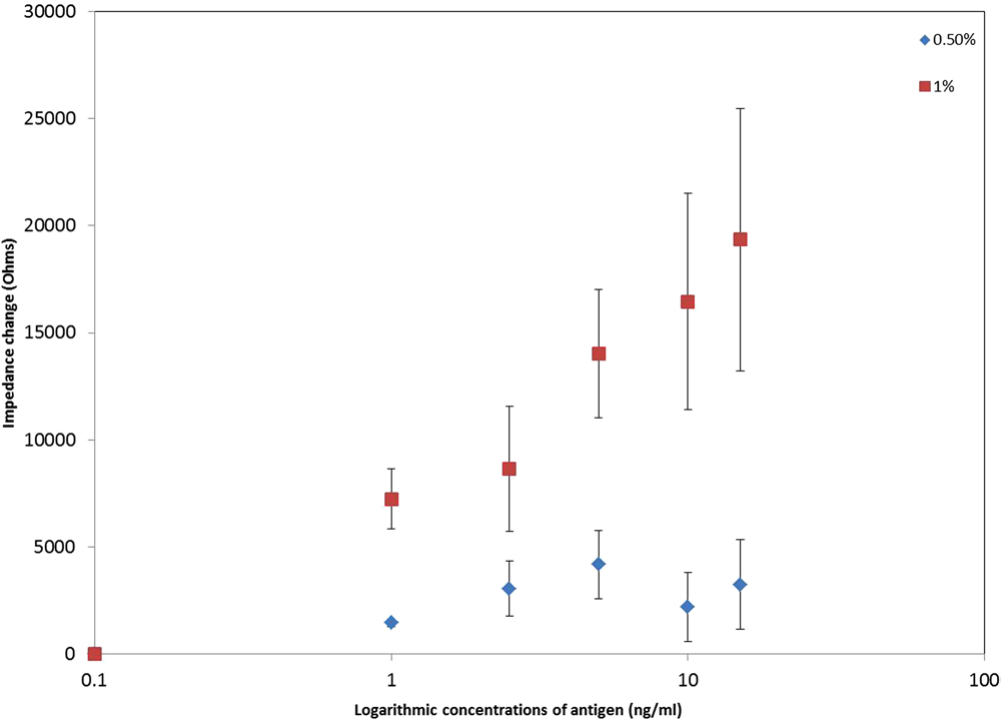
Impedance change in response to increasing concentrations of antigen on 0.5% and 1% ZnO nano-surfaces with 100 ng capture antibody (n = 3).

The nano-surfaces fabricated from different ZnO suspensions were compared by measuring impedance changes with 100 ng capture antibody by adding different concentrations of CRP from 0 to 15 ng/ml at 138 Hz with 10 minutes incubation time. These experimental conditions were selected because, as described above, reproducible differences between the samples were evident at 138 Hz. In addition, from Fig. 6, the absolute impedance measurement have shown to perform reliably with a 10 minutes incubation time. The modulus of the impedance differences (impedance value minus the blank) were employed to plot a calibration curve as shown in Fig. 7. The impedance difference plot shows increasing values of the modulus of the impedance difference with increasing concentrations of CRP for 0.5% and 1% ZnO nano-surfaces. A dose dependence relationship is shown for each plot and can be explained as for Fig. 6 (the opposite slope relating to the fact that Fig. 7 is the modulus of the difference, whereas Fig. 6 shows absolute impedance measurement).

EIS has been shown to be effective tool for probing the interfacial proporties such as the charge transfer and charge seperation^7^. Figure 7 shows that biosensors prepared with 1% ZnO gave higher output for each concentration of CRP compared with 0.5%. The performance of the biosensors is related to the availability of antibody on the surface to bind CRP. Increasing concentrations of antigen binds to a fixed amount of antibody in a dose dependent manner defined by the binding affinity. We speculate that there is increased charge accumulation due to the bound antigen on the surface compared with the antigen in solution. In addition, there will be an increased amount of isolated antigen flowing freely in solution, resulting in a decrease in the charge carriers and hence resistance of the solution. Surfaces fabricated using 1% ZnO showed the highest roughness index implying it has the greatest surface area on which the anti-CRP antibody are captured. The low sensitivity of 0.5% ZnO nano-surafce is likely to be due to the fact that using 0.5% ZnO the PET surface is not fully covered and that the ZnO exists as “islands” of nanocrystals in a modified form due to the ultrasonication. Because of the thickness of the flatter 5% ZnO nano-surface, it was easy to break during the process of protein immobilization. Consequently, the impedance tests were unstable with big error bars and are excluded in Fig. 7.

From Fig. 7, it is evident that the limit of detection for 1% is better than 1 ng/ml. (Further work beyond this study is needed to optimise the biosensors and define the specific limit of detection value in each case.) In human blood the normal level of CRP is defined as being less than 10 mg/L which can rise to over 1000 mg/L in cases of severe bacterial infections. Recently the use of high sensitivity measurements of CRP has been shown to be valuable in determining risk of cardiovascular disease. A high sensitivity CRP test (hsCRP) can measure CRP at concentrations of 0.04 mg/L and levels of hsCRP greater than 3.0 mg/L (in the absence of inflammatory processes) is indicative of a high risk of developing cardiovascular disease. The biosensor described in this paper can measure down to 0.001 mg/L and has plenty of scope to allow for dilution of a small blood sample for presentation to a sensor surface and still be able to measure the small changes in CRP concentration related to risk of cardiovascular disease. The biosensor described has the potential to be developed into a rapid, inexpensive diagnostic test for CRP.

## Conclusion

Zinc oxide nano-surfaces can be created simply and at low cost using a new colloidal dispersion technique, incorporating sonication. Raman spectra of the surface indicated the ZnO crystal surfaces was highly ordered and uniform. Impedance analysis confirmed that the ZnO surfaces were highly reproducible, individual measurements being taken across many days. SEM analysis illustrated differences in the surfaces when three different ZnO suspension concentrations were applied. The highest concentration studied (5% ZnO) showed the smoothest surface whereas the lowest concentration (0.5%) showed incomplete coverage of the substrate. 1% ZnO demonstrated full coverage of the surface and the largest surface area which was quantified by a roughness index. After addition of the antibody to the ZnO surface, Raman spectroscopy confirmed that there was coverage of ZnO surface with the IgG antibody.

At a fixed frequency of 138 Hz, a dose dependent response was observed from 0.1 ng/ml to 15 ng/ml CRP for all 3 concentrations of ZnO (5%, 1% and 0.5%). A limit of detection of less than 1 ng/ml was indicated for concentrations of 1%. 1% ZnO nano-surface showed the best results with the highest sensitivity. This may be explained by the fact that the 1% ZnO nano-surface has the highest roughness index of the 3 concentrations, calculated from Fig. 2, and hence the largest surface area for binding. In contrast, the 0.5% ZnO nano-surface shows incomplete coverage, high variability of particle size and shape and also the lowest sensitivity. Interestingly, during biosensor preparation, the 1% ZnO nano-surface was more stable than 5% during the process of protein immobilisation. The method for fabrication of ZnO nano-surface via colloidal dispersion coupled with ultra-sonication is simple and inexpensive and we consider that this is a promising area for future research. Further work is required to fully characterise the biosensor and to optimise the process of fabricating nano-surface and protein immobilisation to confirm the excellent reproducibility.

## Methods

### Materials

Zinc oxide nanopowders (ZnO, 99.9 + %, 80–200 nm) were purchased from US Research Nanomaterials Inc. Biological molecules: monoclonal mouse anti-human C-reactive protein (4C28 Mab: C6) and human C-reactive protein (CRP) (8C72) were purchased from HyTest Ltd. Phosphate Buffered Saline (PBS, pH 7.3 ± 0.2 at 25 °C was purchased from OXOID Microbiology products. The PBS buffer was diluted to 0.025 M adjusting the pH to 7.4.

### Surface preparation

Accurately weighed quantities of ZnO nano-crystals were added to double deionized water to make a range of concentrations of ZnO suspensions: 0.5%, 1% and 5%. The ZnO suspensions were stirred for one hour and then 1.5 ml aliquots were ultra-sonicated for 7 periods of 20 seconds, at 4 minutes intervals using an exponential microprobe (Soniprep 150) at 30 watts. 200 *μ*l ZnO suspensions were dropped onto clean polyethylene terephthalate (PET) surface. Subsequently, they were dried in oven at 65 °C for 80 minutes and cooled to room temperature and stored in a dry atmosphere with silica gel for up to 2 days.

### CRP Sensor fabrication and test

**Figure 8:**
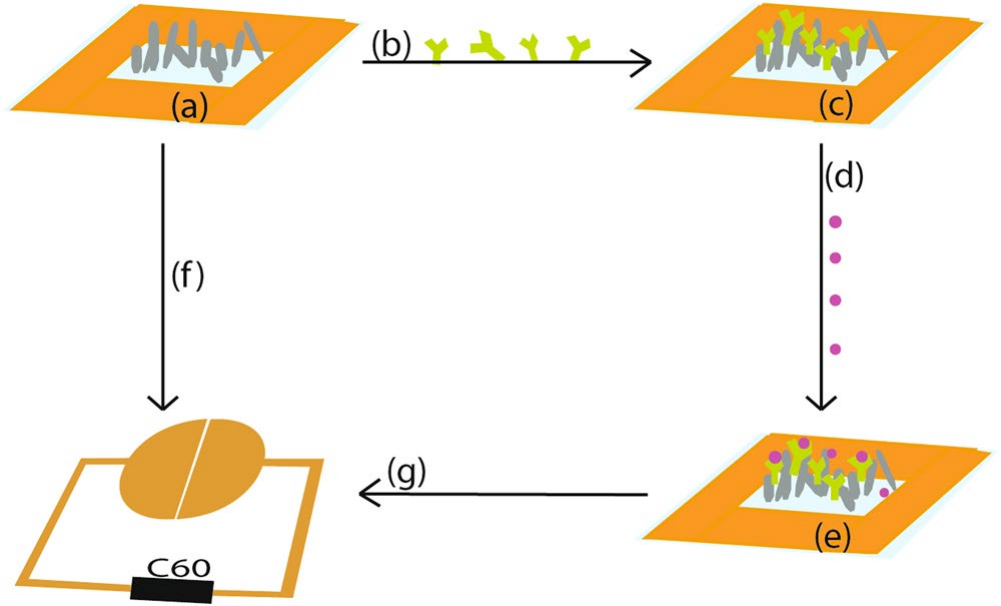
Schematic illustration of biosensor fabrication and sensing: The ZnO sensing area was defined using tape (**a**); then the antibody was added to the ZnO surface (**b**) and dried at 4 °C overnight (**c**); different concentrations of CRP were prepared (**d**) and added to the immobilised antibody on the ZnO surface (**e**); the impedance was measured on a blank surface (**f**) and a surface subjected to the assay protocol (**g**).

The sensing area (10 × 4 mm) of ZnO nanoparticles was defined by tapes (Fig. 8a). Subsequently, 40 *μ*l (100 ng) antibody (C6) was added to the surface (Fig. 8b). The biosensor was then dried in a desiccator with silica gel at 4 °C overnight for 18 hours (Fig. 8c) without wash.

A Cypher Instruments C60 Impedance-Amplitude-Phase Analyser was used to measure the impedance of the nano-crystal surfaces (Fig. 8f). The frequency was scanned from 10 Hz to 4 MHz at a voltage of 2 Vpp, and a DC offset of 0.9 mV, with 300 test points. The impedance plots were analysed using Cypher Graph V1.21.0, Impedance Amplitude and Phase Analyser graphing application software. Impedance spectra of ZnO nano-surfaces were acquired on 24 independent measurements at each of the three concentrations of ZnO used to prepare the surface.

Statistical tests were performed using Minitab. The %CV = (Standard Deviation/Mean*100) was used to compare variation and demonstrate reproducibility within groups. A 2-Sample t-test for between group comparisons was used to prove significant differences, a p value of <0.05 was considered significant.

CRP was prepared at a range of concentrations: 0 (PBS only), 1, 2.5, 5, 10, and 15 ng/ml in PBS (Fig. 8d). 75 *μ*l of each concentration of antigen was added to the biosensor (Fig. 8e) without wash. Following set incubation times the impedance was measured (Fig. 8g) without wash. Impedance measurements were also made at each stage of the assay process, namely: (1) On the nano-crystal surfaces with dry antibody; (2) Instantly, after adding 75 *μ*l of different concentrations of antigen and (3) at 5 minutes intervals until 30 minutes incubation time had passed. In order to plot logarithmic concentrations of CRP, the measurement of PBS buffer only with no CRP (the blank), was defined 0.1 ng/ml CRP (rather than 0 ng/ml). The difference in impedance was derived by subtracting the blank impedance value from each impedance measurement of the CRP assay at the various concentrations.

### Characterization

The morphology of ZnO nano-surface was analysed by Scanning Electron Microscopy (SEM). The samples were coated with a thin layer of Au prior to analysis. A grayscale profile of each image was created using Image J software. Grayscale values along a horizontal line comprising 966 points, were used to produce a’Roughness Index’. A grayscale value (0–255) is a single number that represents the brightness of the pixel. The light areas indicate charge on the surface in the SEM with higher points reflecting electrons and accumulating charge more easily. This means that “deeper” areas do not reflect or accumulate charge as readily and consequently have lower values. Roughness Index was defined as the summation of absolute changes in grayscale values between each two adjacent points. Consequently, the Roughness Index gives an indication of surface area; a larger index means a larger surface area. Grayscale value plots were derived from the images shown in Fig. 1(a,b,c) by placing a horizontal line across the image using ImageJ software, as shown in Fig. 2.

Raman spectroscopy was used to distinguish the chemical compositions of the ZnO nano-crystal surfaces. A XploRA Raman spectrometer from Horiba, equipped with a confocal microscope, was used. The Raman signals were collected in a range of 0–3500 cm^−1^ using a 785 nm red laser excitation. The laser beam was focused on the sample using objective magnification of 50×.
